# Alterations of Cortisol and Melatonin Production by the Theca Interna Cells of Porcine Cystic Ovarian Follicles

**DOI:** 10.3390/ani12030357

**Published:** 2022-02-01

**Authors:** Yusheng Qin, Jiahua Bai, Jiage Dai, Jianhui Zhou, Taipeng Zhang, Silong Zhang, Xiaoling Xu, Yan Liu

**Affiliations:** 1 Institute of Animal Husbandry and Veterinary Medicine, Beijing Academy of Agriculture and Forestry Sciences, Beijing 100097, China; blackberrysheng@163.com (Y.Q.); bai_jiahua@126.com (J.B.); ztp5331365@163.com (T.Z.); zhang_silong@126.com (S.Z.); xu_xiaoling1980@163.com (X.X.); 2 College of Animal Science and Technology, China Agricultural University, Beijing 100193, China; djg730@126.com; 3 Guilin Animal Husbandry Station, Guilin 541000, China; zjh1766@163.com; 4 College of Life Sciences and Food Engineering, Hebei University of Engineering, Handan 056038, China

**Keywords:** pig, follicular cyst, cortisol, melatonin, steroid hormones

## Abstract

**Simple Summary:**

The mechanism of follicular cyst formation is largely unknown but changes in follicular composition are known to be involved. In particular, there is abnormal hormone secretion in cystic follicles. Here, we found there was disruption of hormone secretion in the fluid of cystic follicles in sows. The glucocorticoid receptor was highly expressed, and the melatonin receptor was weakly expressed in cystic follicles compared with control follicles. Thus, secretion of steroid hormones in cystic follicles is disrupted and disturbances in signaling via cortisol and melatonin are involved in the development of follicular cysts in sows.

**Abstract:**

(1) Background: Cortisol and melatonin (MT) act in regulating follicular development. We hypothesized that abnormal levels of cortisol, MT, and steroids in theca interna cells might be involved in the development of follicular cysts in sows. (2) Methods: To test this hypothesis, we measured the mRNA levels of enzymes involved in steroid hormone synthesis, the glucocorticoid receptor (GR), and melatonin receptors (MTRs) in theca interna cells of cystic and normal porcine follicles. (3) Results: The concentrations of estradiol, progesterone, and cortisol were greater in cystic follicles than in control ones (*p* = 0.034, *p* = 0.020, *p* = 0.000), but the concentration of MT was significantly lower (*p* = 0.045). The levels of *GR, 11β-HSD1*, and *11β-HSD2* were higher in cystic follicles than in control l follicles. MT types 1 and 2 were significantly lower in cystic follicles (*p* < 0.05). The mRNA expression levels of genes encoding the steroid hormone synthesis enzymes, steroidogenic acute regulatory protein (*StAR*), recombinant cytochrome *P45011A1* (*CYP11A1*), and 3β-hydroxysteroid dehydrogenase (*3β-HSD*) in theca interna cells of cystic follicles were significantly higher than in control follicles. Thus, there was disruption of hormone secretion in the fluid of cystic follicles in sows. (4) Conclusions: The levels of steroid hormones, cortisol and MT are disrupted in porcine cystic follicles.

## 1. Introduction

A follicular cyst is a kind of ovarian cyst [1] and is a major factor causing infertility in sows, goats, and cattle [2,3,4]. Follicular cysts are associated with 10% of cases of reproductive failure in sows [2,5]. This disease impairs their reproductive performance and causes serious economic losses to pig breeding farms [6]. It is generally believed that animal stress, mismanagement, infectious diseases, and other factors that lead to an abnormal cortisol increase and endocrine disorders are major factors in follicular cyst formation [4,7,8]. The mechanisms are largely unknown, but changes in follicular composition are known to be involved [9,10,11].

The adrenal cortex is the only organ involved in glucocorticoid synthesis, and cortisol is the major glucocorticoid product [12]. This hormone is distributed throughout the body via the bloodstream and enters cells to play its physiological role. Melatonin (MT) is an indoleamine hormone mainly produced by the pineal gland of mammals and is distributed in the pineal gland and in several other organs, such as the ovary and testes [13,14]. The biological functions of MT are mediated by its two high-affinity G-protein-coupled receptors, MT1 and MT2 [15]. MT can act on the hypothalamic–pituitary–ovarian axis (HPO) by regulating the secretion of hypothalamic gonadotropins, which can also directly bind to the ovarian granulosa cells [16,17]. MT in the ovary can be derived from the systemic blood circulation, or synthesized by the granular cells, including the cumulus granulosa cells and oocytes [18]. Circulating MT can be absorbed by the ovaries, but ovarian follicles also have the ability to synthesize and secrete MT [19]. The level of MT is higher in follicular fluid (FF) than in the blood [20], and its concentration in FF rises significantly as follicles mature [21]. MT affects reproductive physiology by modulating sex steroid secretion at various phases of folliculogenesis, mainly mediated via its MT1 and MT2 receptors [22]. Therefore, MT has important paracrine effects in the female reproductive system. A significantly decreased expression was observed for the MT2 receptor in PCOS induced by letrozole in rats [23], and the mRNA expressions of MT1 and MT2 decreased in the theca cells of cystic follicles. Cortisol and MT are involved in regulating follicular development and maintain the follicular microenvironment through glucocorticoid receptors (GRs) and melatonin receptors (MTRs), respectively [24,25].

The cellular levels of the GR and 11β-hydroxysteroid dehydrogenase (11β-HSD) regulate the concentrations and effects of glucocorticoids in tissues. As a major regulator of cortisol metabolism, 11β-HSD is expressed as two isoenzymes, 11β-HSD1 and 11β-HSD2 [26], which regulate follicular development by changing the concentration of cortisol during follicular development and can act in the development of endocrine diseases. 11β-HSD1 converts non-bioactive cortisone to active cortisol, thereby regulating cortisol levels available to intracellular GRs, while 11β-HSD2 converts cortisol to cortisone to protect the mineralocorticoid receptor from undue occupation by cortisol [27]. Under the regulation of 11β-HSD, cortisol in the FF is converted to cortisone and participates in the regulation of follicular development. Blood cortisol levels are abnormally elevated in sows with follicular cyst formation in response to heat stress [28]. The level of cortisol in the FF of cystic bovine follicles is significantly higher than that in normal follicles, and the expression of 11β-HSD1 is significantly increased in the granulosa cells of such follicles [29]. These findings suggest the involvement of cortisol and its metabolic enzymes in the occurrence of follicular cysts in cattle, but there are few studies on spontaneous cystic follicles in sows.

Cortisol and MT levels regulated by the circadian rhythm are out of synchrony. Thus, cortisol secretion peaks during the day, whereas MT peaks at night. MT may play an important role in metabolic diseases, and its absence in pinealectomized animals causes the development of ovarian cysts via the altered synthesis of luteinizing hormone (LH) and follicle stimulating hormone [13]. The acute lowering of cortisol secretion stimulates MT secretion. When MT is low in the serum, it leads to increased cortisol secretion, and the administration of exogenous prolonged-release MT can rectify cortisol production.

Follicular theca interna cells provide structural support for follicles and secrete precursors for steroid synthesis by the granulosa cells. Here, we speculated that porcine theca interna cells might show abnormal expression of steroid synthetase activities and signaling of cortisol and MT, leading to the formation of cystic follicles.

## 2. Materials and Methods

### 2.1. Ethics

The study was conducted at the Beijing Academy of Agriculture and Forestry Sciences, and use of animals in the experiments was approved by the Ethical Committee of Beijing Academy of Agriculture and Forestry Sciences (SYXQ-2012-0034).

### 2.2. Collection of Ovaries

Gilts (crossbred Landrace × Large white 110–130 kg body weight, aged 200 to 220 days), were used. Ovaries from spontaneous follicular cysts of sows (*n* = 5) and control follicles (*n* = 5) were collected from a local abattoir and transported to the laboratory within 2 h in pre-warmed phosphate-buffered saline (PBS; 37 °C) with Pen-Strep antibiotic solution (Biological Industries, Beit HaEmek, Israel). Follicular cysts were diagnosed on the basis of macroscopic characterization (>20 mm diameter, fluid-filled with smooth thin and translucent walls, and the absence of corpora lutea on the ovaries). Normal control follicles (~4–6 mm in diameter) with no gross morphological abnormalities were used as controls.

### 2.3. Collection of Follicular Fluid and Theca Interna

Ovaries were washed two to three times with PBS in a 100 mm Petri dish. Individual follicles were dissected carefully from the ovarian stroma using forceps and scissors. After making a small incision with a scalpel, two blunt-tipped forceps were used to peel off the outer membrane from the incision, which left the intact theca interna containing FF. This was carefully aspirated from cystic and control isolated follicles with a syringe. The FF was centrifuged at 1000× *g* for 5 min and stored at −80 °C until hormone measurements. The theca interna was collected by modifying the method of Hatzirodos [30]. Follicles were dissected, and granulosa cells were aspirated and scraped from each follicle with a Pasteur pipette, and washed at least three times to remove other cell types, and the granulosa cells were discarded. The theca interna was then dissected from the follicle wall under a stereomicroscope in PBS. The theca interna was then frozen in liquid nitrogen and stored at −80 °C for RNA extraction and mRNA analysis.

### 2.4. Steroids, Cortisol, and Melatonin Assays

Estrogen (GEL4598-A) was measured in FF using enzyme-linked immunosorbent assays (ELISAs) for pigs (Gene Lab Biotechnology Co., Ltd., Beijing, China), according to the manufacturer’s instructions; the intra- and inter-assay coefficients of variation for serum were 4.1–6.8% and 6.7–9.4%, respectively. Progesterone (GEL4686-A) was measured in FF using ELISA for pigs (Gene Lab Biotechnology Co., Ltd., Beijing, China), according to the manufacturer’s instructions. The intra- and inter-assay coefficients of variation for the serums were 2.9–4.8% and 6.8–9.2%, respectively. The level of MT in FF was measured using a specific ELISA kit (RE54021, IBL International Gmbh, Hamburg, Germany). For the assays, the sensitivity was 1 ng/mL, and the intra- and inter-assay coefficients of variation were 5.2–12.2% and 5.1–14.9%, respectively. The concentration of cortisol was measured using ^125^I-labeled radioimmunoassay kits (S10940097, Beijing North Biotechnology Institute, Beijing, China), according to the manufacturer’s instructions. For the assays, the sensitivity was 2 ng/mL, and the intra- and inter-assay coefficients of variation were <10% and <15%, respectively.

### 2.5. Quantitative Reverse Transcription Polymerase Chain Reaction (RT-qPCR)

Total RNA was isolated using RNAzol reagent (RNAzol RT reagent, rn190; Molecular Research Center, Cincinnati, OH, USA). A NanoDrop 2000c spectrophotometer (Thermo Fisher Scientific Inc., Wilmington, DE, USA) was used for qualitative analysis. The PCR primers for genes were designed by NCBI. All primer sequences, accession numbers, product length and primer positions for qPCR are listed in Table 1. Primer sequences were synthesized by Shanghai Bioengineering Co., Ltd. Quantitative amplification of cDNA was performed in 0.2 mL PCR tubes using iScript advanced cDNA synthesis kits (Bio-Rad Laboratories, Hercules, CA, USA). Amplification efficiencies of the primer set candidates can then be verified experimentally and their specificity confirmed by melt-curve analysis and agarose gel electrophoresis of RT-qPCR amplification products. The qPCR was performed using a Bio-Rad (Bio-Rad Laboratories, Hercules, CA, USA) Chrome 4 Real-Time qPCR System. The qPCR mix (10 μL) included 5 μL of SYBR green premix, 0.3 μL of each forward and reverse primer (10 μmol/L), 4 μL of cDNA and 0.4 μL of dH_2_O. The qPCR conditions were as follows: 2 min denaturation at 95 °C, 40 cycles of PCR for the quantitative analysis (95 °C for 5 s and 60 °C for 30 s), one cycle for the melting curve analysis (95 °C for 5 s, 60 °C for 1 min, 95 °C for 1 s) and cooling at 4 °C. The relative expression level for each gene was calculated using the 2^−ΔΔCT^ method. The qPCR analysis was performed three times for each group sample. We defined the gene expression cut-off as a mean Ct value of 35. *GAPDH* was used as the reference gene for *GR*, *11β-HSD*, *MT1*, *MT2*, *LHCGR*, *StAR*, *3β-HSD*, *CYP11A1*.

### 2.6. Data Analysis and Statistics

Data from control and cystic follicle groups were analyzed using two-tailed Student’s *t* tests with IBM SPSS Statistics for Windows version 20.0 (IBM Corp., Armonk, NY, USA). All data are presented as the mean ± standard deviation. A *p* value of <0.05 was considered statistically significant. Statistical significance was evaluated using data from at least three independent experiments.

## 3. Results

### 3.1. Hormone Concentrations in Follicular Fluid

**Figure 1:**
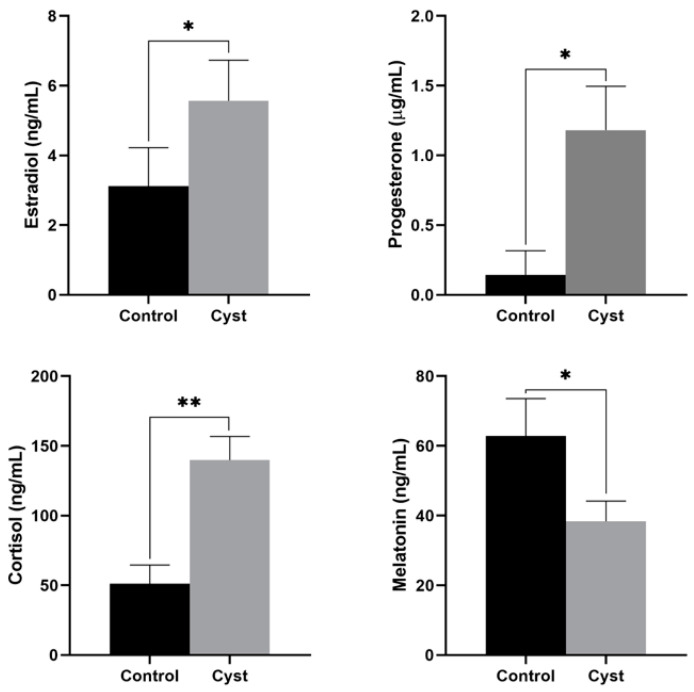
Concentrations of estradiol, progesterone, cortisol, and melatonin in the follicular fluid of cystic and control follicles. * Indicates statistically significant (*p* < 0.05); ** Indicates statistically extremely significant (*p* < 0.01).

The concentrations of estradiol, progesterone, cortisol, and MT in the FF of cystic and control follicles are shown in Figure 1. The estradiol concentration was significantly higher in the cystic follicles (*p* = 0.034). The progesterone concentration was higher in cystic follicles than in control follicles (*p* = 0.020). The concentration of cortisol in cystic FF was much higher than that in control follicles (*p* = 0.000). However, the concentration of MT was significantly lower (*p* = 0.045).

### 3.2. Relative mRNA Levels of GR, 11β-HSD, MT Receptor and Steroidogenic Enzymes

**Figure 2:**
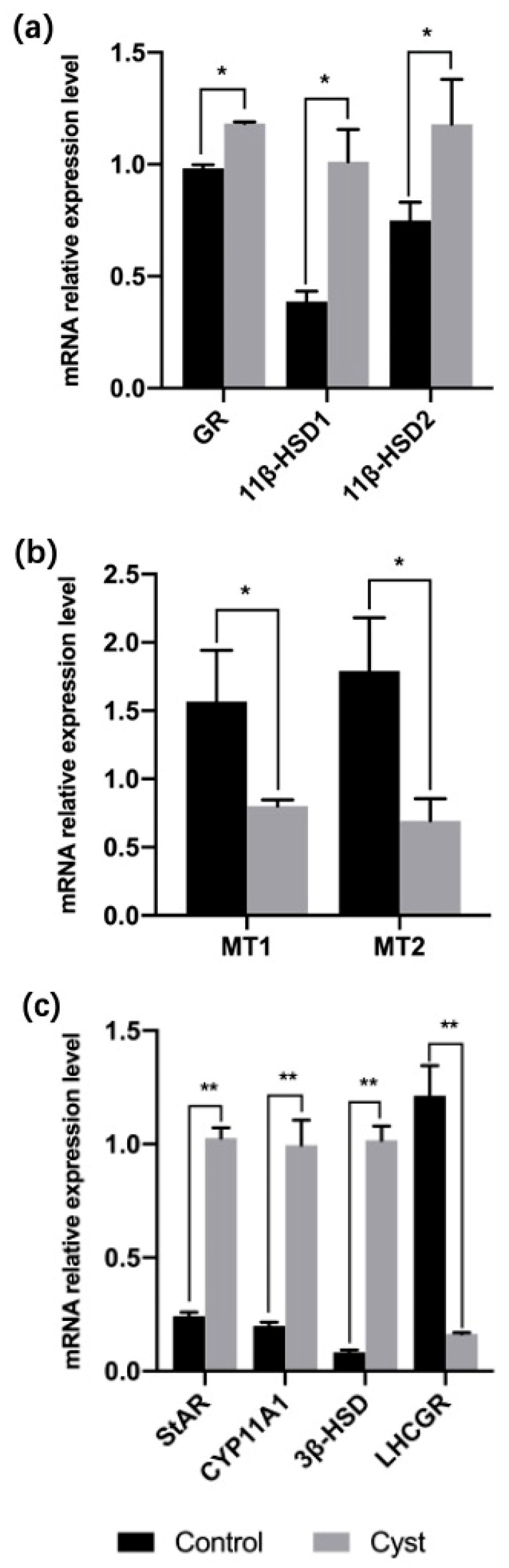
(**a**) mRNA levels for *GR* and *11β-HSD1/2* in the theca interna of cystic and control follicles. (**b**) mRNA levels of MT receptors in the theca interna of cystic and control follicles. (**c**) mRNA levels of steroidogenic enzymes and *LHCGR* in theca interna of cystic and control follicles. * Indicates statistically significant (*p* < 0.05); ** Indicates statistically extremely significant (*p* < 0.01).

The expression levels of *GR* mRNA in the theca interna of cystic follicles were significantly higher than those in control follicles (*p* = 0.016). Moreover, *11β-HSD1* and *11β-HSD2* mRNA levels were higher in cystic follicles than in control follicles (*p* = 0.011; *p* = 0.026; Figure 2a). The transcription levels of MT1 and MT2 were lower in cystic follicles than in control follicles (*p* = 0.025; *p* = 0.011; Figure 2b). Next, we measured the mRNA levels of *StAR* and steroid hormone synthase genes by RT-qPCR (Figure 2c). The mRNA expressions of *StAR*, *CYP11A1* and *3β-HSD* in theca interna cells of cystic follicles were significantly higher than in control follicles (*p* = 0.000; *p* = 0.005; *p* = 0.001). Expression of *LHCGR* in the theca interna of cystic follicles was significantly lower than that in control follicles (*p* = 0.005).

## 4. Discussion

As the basic unit of ovarian structure and function, a follicle is not only the site of oogenesis but also the site of steroid hormone synthesis and secretion. Normal follicular development requires subtle and precise regulation by interactions between these hormones and a complex signaling network [31]. Disruption of hormone secretion can lead to ovarian diseases and impair the reproductive performance of animals [32]. Cortisol and MT are involved in regulating follicle development and maintaining the follicular microenvironment [33,34,35]. The abnormal change of concentration of these molecules causes ovarian dysfunction [13,29].

We found that the concentration of cortisol in the FF of cystic follicles was significantly higher than in control follicles. Moreover, sows with low-quality cumulus-oocytes complex have FF with a higher concentration of cortisol [36]. Cortisol is higher in the FF of spontaneous or adrenocorticotropic hormone-induced follicular cysts in cattle [29], which is consistent with these results. Stressors induce elevated cortisol levels and suppress the HPO axis activity [37]. Stress-induced increases in adrenal glucocorticoids cause an increase that contributes to the hypothalamic suppression of reproductive function [38]. Cortisol affects follicular function as determined by the amount of GR, the intracellular concentration of glucocorticoids, and the activity of 11β-HSD during follicular development [39]. In this study, the mRNA expressions for *GR* and *11β-HSD1/2* were higher than in normal follicles. Cortisol concentrations in the FF and *11-HSD1* mRNA are significantly elevated in human patients with polycystic ovarian syndrome (PCOS); increased 11-HSD1 expression is the major cause of increased cortisol concentrations in the FF of such patients [40]. Additionally, there might be disruption of the internal follicular environment, which could also be a factor in the high expression of 11*β*-HSD2 [41]. In theca interna cells of cattle, *GR* expression was higher in spontaneous cystic ovarian follicles than in normal tertiary follicles [42]. The increase in *GR* and *11-HSD* expression in cystic follicles could be related to the formation of follicle cysts in sows.

High concentrations of cortisol affect the synthesis and secretion of MT [43], but also influence the physiological function of MT in follicles by suppressing the expression of MT receptors. MT can act on the HPO axis by regulating the production of hypothalamic gonadotropins, which can also directly bind to ovarian granulosa cells to exert effects on the HPO axis [15]. Low MT levels are linked to ovarian problems, MT levels in the FF of women with PCOS are notably lower than in healthy women [13]. Consistent with the results of this study, MT levels in the cystic follicles of gilts were lower than in control follicles. A significantly decreased expression was observed for the MT2 receptor in PCOS induced by letrozole in rats [23], and the mRNA expressions of MT1 and MT2 decreased in the theca cells of cystic follicles in the present study.

The levels of estrogen, progesterone and steroidogenic enzymes expression increased. Estrogen excretion by sows with large cystic follicles was relatively high [44], and cold stress increased progesterone and cortisol levels [45]. The inhibited gene expression of steroidogenic enzymes (*Cyp11a1*, *StAR* and *3β-HSD*) reduced the production of progesterone and 17β-estradiol [46]. Glucocorticoids regulate the expression of *StAR* through the GR and affect the synthesis of steroid hormones [47]. Acute stress induced by capture, short confinement, or anesthesia results in significant elevation of plasma cortisol and increased mRNA post-stress levels of *StAR* and *CYP11A1* [48]. Here, during the formation of porcine follicular cysts, excess cortisol might have affected the mRNA expressions of *StAR*, *CYP11A1*, and *3β-HSD*, resulting in abnormally elevated levels of progesterone in FF. LH is a necessary factor that triggers ovulation via the *LHCGR* [42]. Glucocorticoids influence the gonadal responsiveness to LH and the expression of *LHCGR* [49]. Enhanced secretion of cortisol decreases *LHCGR* content in follicles [50]. Here, the mRNA expression level of *LHCGR* was significantly decreased in the theca interna cells of cystic follicles, consistent with findings that the concentration of corticosterone in rat increased under constraining stress and that the expression of *LHCGR* decreased significantly [48]. How elevated cortisol induces follicular cysts remains to be determined.

## 5. Conclusions

The levels of steroid hormones, cortisol and MT were clearly disrupted in the cystic follicles of gilts. Molecular alterations of steroid hormone synthases, GR, LHCGR, and the MTR might be involved in this pathology.

## Figures and Tables

**Table 1:** Primers for PCR amplification.

Gene	Primer Sequence	Primer Positions	Accession Numbers	Product Length
*GAPDH*	F: TGAAGGTCGGAGTGAACGGATT	105–126	NM_001206359.1	120
	R:CCATGTAGTGAGGTCAATGAAGG	224–201		
*StAR*	F:GACTTTGTGAGTGTGCGCTG	547–566	NM_213755.2	108
	R:AGCTCTGATGACCCCCTTCT	635–654		
*3β-HSD*	F:GTTCTCCAGAGTCAACCCCG	651–670	NM_001004049.2	112
	R:GTTCTCCAGAGTCAACCCCG	743–762		
*CYP11A1*	F:CCGCTCAGTCCTGGTCAAAG	24–43	NM_214427.1	145
	R:GTCACCAGGAGAGGGGATCT	149–168		
*LHCGR*	F:GCTGATTTCCCTGGAGCTGA	594–613	NM_214449.1	124
	R:ACTAGGCAGGGCCTGTAGTT	698–717		
GR	F:GTGATGGGAAGTGACCTGGG	292–311	NM_001008481.1	231
	R:CTGACCCTTCACATTCGGCT	503–522		
*11β-HSD1*	F:CACGCTCTGTATCCTCGGTC	630–649	NM_214248.3	201
	R:TCCAGGATCTTCCTCCCTGG	811–830		
*11β-HSD2*	F:GGAGTTGGATAGCCCTGGTG	342–361	NM_213913.1	173
	R:TGTTGTGGCCTGCATTGTTG	495–514		
*MT1*	F:ACAAGAAGCTGAGGAACGCA	209–228	XM_021078041.1	207
	R:TGATGGCAATTCCCGCGATA	396–415		
*MT2*	F:CCAGAACTTCCGCAGGGAAT	738–757	XM_021063941.1	126
	R:CTAACCTCGGGGAGAGCTTG	844–863		

## Data Availability

The data presented in this study are available on request from the corresponding author. The data are not publicly available due to data are still being processed to produce other papers.
